# Factors Associated with American Indian Cigarette Smoking in Rural Settings

**DOI:** 10.3390/ijerph8040944

**Published:** 2011-03-30

**Authors:** Felicia Hodge, Karabi Nandy

**Affiliations:** 1 School of Nursing, University of California, Los Angeles, P.O. Box 951702, Los Angeles, CA 90095, USA; E-Mail: knandy@sonnet.ucla.edu (K.N.); 2 School of Public Health, University of California, Los Angeles, P.O. Box 951772, Los Angeles, CA 90095, USA

**Keywords:** American Indian, tobacco, cigarette smoking, rural populations, prevalence, patterns of smoking

## Abstract

**Introduction:**

This paper reports on the prevalence, factors and patterns of cigarette smoking among rural California American Indian (AI) adults.

**Methods:**

Thirteen Indian health clinic registries formed the random household survey sampling frame (N = 457). Measures included socio-demographics, age at smoking initiation, intention to quit, smoking usage, smoking during pregnancy, health effects of smoking, suicide attempts or ideation, history of physical abuse, neglect and the role of the environment (smoking at home and at work). Statistical tests included Chi Square and Fisher’s Exact test, as well as multiple logistic regression analysis among never, former, and current smokers.

**Results:**

Findings confirm high smoking prevalence among male and female participants (44% and 37% respectively). American Indians begin smoking in early adolescence (age 14.7). Also, 65% of current smokers are less than 50% Indian blood and 76% of current smokers have no intention to quit smoking. Current and former smokers are statistically more likely to report having suicidal ideation than those who never smoked. Current smokers also report being neglected and physically abused in childhood and adolescence, are statistically more likely to smoke ½ pack or less (39% *vs.* 10% who smoke 1+ pack), smoke during pregnancy, and have others who smoke in the house compared with former and never smokers.

**Conclusion:**

Understanding the factors associated with smoking will help to bring about policy changes and more effective programs to address the problem of high smoking rates among American Indians.

## 1. Introduction

American Indians (AIs) report higher smoking rates than any other racial/ethnic group in the U.S. with rates more than double that of the general population [1,2]. Extremely high smoking rates have been reported among American Indian groups since the 1980s [3]. While the general population has responded to cessation programs and policies and has experienced reductions in overall smoking rates, American Indian smoking patterns continue unabated. This situation calls for targeted studies that address the uneven effects of intervention efforts for American Indians.

According to a recent Centers for Disease Control and Prevention (CDC) study, 24.3% of Americans are current smokers. Further, findings indicate the populations most affected by tobacco use are vulnerable minority populations [4]. The 2010 CDC Morbidity and Mortality Weekly Report [5] (based on the 2008 Behavioral Risk Factor Surveillance System) identified the states where the general smoking prevalence was the highest as West Virginia (26.6%), Indiana (26.1%), and Kentucky (25.3%); the lowest prevalence of smoking is found in Utah (9.2%), California (14%) and New Jersey (14.8%). In contrast, 30–40% of American Indians in the U.S. are current smokers. The regions with the highest smoking rates among American Indians include Alaska (40%) [6], the Northern Plains (44%) [7] and California (40%) [3]; the lowest prevalence of American Indian smokers occurs in the Southwest (21%) [8].

The disparity in smoking rates among American Indians is alarming on several levels. Firstly, not only are American Indian smoking rates nearly double that of the general population, but the states where American Indian smoking rates are reported to be the highest do not match up with the states identified as having high smoking rates among the general population.[9] American Indian reservations are generally located west of the Mississippi River and large concentrations of American Indians reside in western cities where the Federal government established Indian relocation sites (e.g., Los Angeles, San Francisco, Seattle, *etc.*) [9]. It is possible that large-scale smoking cessation intervention studies targeting the general population in states with high general smoking rates may not be reaching American Indian smokers where they reside and where concentrations of American Indian smokers are located. Secondly, what is known about American Indian patterns of smoking (high smoking rates among both males and females, few numbers of cigarettes, smoking on a daily basis, *etc.*) differs from the patterns of smoking for the general population (heavy smoking primarily among males). Therefore, general cessation programs are likely ill-fitted for this group, essentially marginalizing American Indians and doing little to reduce the smoking disparity. Thirdly, culturally-sensitive and appropriate projects have been few and far between, as only a limited number of cessation programs have been funded for specific tribal cultures and fewer have been evaluated and scientifically measured in terms of effectiveness and sustainability. Consequently, there is a need for culturally-appropriate cessation programs that utilize better metrics, design, frameworks, reporting and policies for American Indian populations.

Gaining a better understanding the factors associated with smoking behaviors will help to bring about targeted interventions, policy changes and more effective programs to address the problem of high smoking rates among American Indians. This paper reports on the prevalence, factors and patterns of cigarette smoking among rural California American Indian adults.

## 2. Methods

*Recruitment:* The Indian Health Service (IHS) reports that the majority of rural American Indians utilize Indian healthcare clinics for the majority of their healthcare needs [10]. Therefore, rural Indian clinics were targeted to identify and access the population in this study. Through a process of collaboration and agreement with IHS healthcare clinic sites who agreed to participate in the study, American Indian adult residents of rural Indian households in California were recruited for this study.

Thirteen Indian health clinic registries located on or near rural reservations in California formed the sampling frame for a random household survey. Inclusion criteria included (a) being age 18 years or older, (b) residing on one of the 13 participating reservation/rancheria sites, and (c) being a client (user) of one of the local Indian healthcare clinics within the past five years. All adult members of the randomized households were approached by a research staff member in person, via telephone call or mailing requesting their participation in the study. Research staff explained the nature and purpose of the study at this recruitment phase. Care was taken to fully inform each adult participant that his or her involvement in the study was voluntary and confidential. Site approvals were obtained through letters of agreement from each site and/or clinic. Institutional Review Board approvals were obtained from the University of California at Berkeley, the University of California at San Francisco, the University of Minnesota and the Indian Health Service. Participant consent was obtained according to the Protection of Human Subjects protocol as approved by the sponsoring university. Participants were read and signed a consent form. Incentives were provided in the form of decorative bags and mugs.

*Sample:* The study sample was comprised of American Indians aged 18 years and older who were active clients of local Indian healthcare clinics (defined as clinic user within the past 5 years), residing in California within the service areas of the local rural reservation/rancheria sites and were members of the selected study households. Five hundred adult American Indians were recruited into the study and 457 agreed to participate.

*Design:* The study employed a cross-sectional randomized household survey design at California rural/reservation sites. American Indian households were identified through the IHS clinic health registries, which formed the sampling frame for a random household survey. Households were then randomly selected and a self-administered questionnaire was administered to all adult members of participating households. Fifteen healthcare clinics sites were approached and 13 agreed to participate in the study.

*Data Collection Method:* The 1998 Behavioral Risk Factor Surveillance System (BRFSS) was modified to include questions relating to abuse and neglect and tribal identity (affiliation and blood quantum). All selected household member participants provided responses to the survey in person. Sixty percent of participants chose to complete the survey in their homes; the remainder completed the questionnaire at a private clinic meeting room.

*Measures:* The survey instrument was a 60 minute questionnaire to measure the following:

Socio-demographic characteristics: Gender, tribal affiliation, degree of Indian blood (grouped into cohorts, <25%, 25%, 50%, 75%, and 100%), employment, income (annual average) and educational attainment (<high school degree and ≥high school degree) were collected. Age was grouped into cohorts (18–24, 25–39, 40–54 and 55+). Marital status was dichotomized into married (or living with someone) and single (divorced/separated/widowed/single).

Perceived general health: Participants were asked, “In general, would you say your health is: excellent, very good, good, fair or poor?” This measure was categorized based on participant response (very good = excellent/very good, good, and poor = fair/poor).

Perceived wellness status: Participants were asked, “Wellness includes feeling good and taking care of yourself physically, emotionally, mentally and spiritually. How would you rate your own wellness: excellent, good, fair or poor?” This measure was categorzed into two groups (good = excellent/good, and poor = fair/poor).

High-risk behaviors: Measures for high-risk behaviors included use of alcohol during lifetime. Data on drug usage was not reported in this paper due to the low response rate and concern that responses were not truthful due to fear, stigma, legal reasons and family issues.

Psycho-social: Measures included self-reported history of childhood, adolescent or adult physical or sexual abuse and neglect. Information on suicidal ideation and suicide attempts was also collected.

Smoking status: Current smokers were categorized as those who smoked at least 100 cigarettes in their entire life and currently smoke. Former smokers were categorized as those who smoked at least 100 cigarettes in their entire life and do not currently smoke. Those who had not smoked at least 100 cigarettes in their entire life and did not currently smoke were considered never smokers [11].

Patterns of smoking use and cessation: Measures included average number of cigarettes smoked in a day, age when first whole cigarette was smoked, intention to quit smoking (in next 30 days), smoking during pregnancy, smoking at work, smoking in home and number of smokers in the home.

*Data analysis:* Smoking status (current, former and never smokers) was assessed for this sample of American Indians. Differences among these categories were studied under the broad classes of demographics, perception of wellness, health status, high-risk behaviors and history of neglect and abuse. Chi-square tests were used to assess statistical differences between categorical variables by smoking status, whereas generalized linear models were used to assess the differences in continuous outcomes by smoking status. Further comparisons between current and former smokers were assessed in terms of their smoking behaviors and environment using chi-square and t-tests. Backward multiple logistic regression analysis was used to create a predictive model for smoking status (current and former); predictors included variables that were associated with current/former smoking status at the 0.15 level in preliminary analyses; covariates were retained if they were significant at the 0.10 level. Goodness-of-fit of the model was assessed by the Hosmer-Lemeshow test, as well as by area under the curve (AUC) of the associated receiver operating characteristic (ROC) curve. All statistical analyses were performed with Statistical Analysis Program (SAS/STAT).

## 3. Results

*Demographic characteristics:* The sample consisted of 457 rural American Indian adults in California. Three quarters of the sample was female (n = 317) and the average age was 44.79 (sd = 15.88), ranging from 28 to 75 years old. Nearly a quarter (21.8, n = 93) had less than a high school education and 62.8% (n = 260) were employed. The average annual household income was $24,468.84 (sd = 19,931.33) and 52.6% (n = 219) were married or living together, whereas 47.4% (n = 197) were single, separated, divorced, or widowed. The majority of households (82%, n = 349) reported having a telephone in their homes. Eighty-seven percent (n = 371) were enrolled in a tribe and 43% (n = 183) of the sample reported that they had 50% or more Indian blood quantum.

Table 1 reports the demographic characteristics of American Indian adults by smoking status. Four hundred and twenty-six out of 457 participants in the sample (93% response rate) answered questions about their smoking status. Smoking status was determined based on the three categories of current smoker, former smoker and never smoker. Among those that responded, 38% (n = 163, 95% CI: (0.33, 0.43)) were current, 27% (n = 114, 95% CI: (0.23, 0.31)) were former and 35% (n = 149, 95% CI: (0.30, 0.40)) were never smokers. Current smokers were younger (average age 40 years) compared to former smokers (average age 51 years) and never smokers (average age 44 years) (p-value < 0.0001). They were also more likely to be married (60%, n = 95) compared to their former/never smoker counterparts (p-value = 0.07).

*Health and Psycho-social:* Regarding health issues, 21% (n = 88) perceived their general health status to be fair to poor while 38% (n = 163) perceived their general health status to be average. On the other hand, 26% (n = 112) perceived poor wellness. A higher proportion of males was more likely to be current (29%, n = 47) or former smokers (30%, n = 34) than never smokers (18%, n = 27, p-value = 0.04). A significant proportion of former smokers (73%, n = 83) perceived their general health to be average to poor compared to current (55%, n = 90) and never (53%, n = 78) smokers (p-value = 0.002).

A fifth of the sample reported history of neglect or abuse at some stage of their life. Significantly higher proportions of former smokers reported history of neglect in their childhood (23%, n = 25) and adolescence (26%, n = 25) compared to current or never smokers (p-value = 0.02 and 0.01 respectively). Current smokers were more likely to have experienced physical abuse in their lifetime than former or never smokers. For example, in childhood and adolescence a significantly higher proportion of current smokers experienced physical abuse (22%, n = 33; and 18%, n = 26, respectively) compared to lower levels of abuse in former or never categories (p-value = 0.0008 and 0.0003, respectively). Current smokers also reported higher proportions of sexual abuse in their lifetime compared to those of former or never smokers, although not statistically significant.

Of note, significant proportions of both current (24%, n = 38) and former (24%, n = 27) smokers reported having experienced suicidal ideation (p-value = 0.03). Current and former smokers were also statistically more likely to drink alcohol.

*Environment:* Table 2 assesses the differences between current and former smokers by smoking behaviors and smoking environment. The age of initiation to smoking for both groups was the same, 14 years. However, comparisons of smoking environment, such as number of smokers in the house and whether smoking was allowed in the house, showed significant differences between current and former smokers (p-values < 0.0001 on all measures). Seventy percent of former smokers (n = 70) reported no smokers in their house compared to only 5% (n = 8) of current smokers. In fact, almost half the current smokers had two or more current smokers in their house compared to only 9% among former smokers. Half of current smokers said smoking was allowed in their home compared to 23% of former smokers. A similar pattern was observed for whether visitors were allowed to smoke in their homes.

Only a quarter of current smokers said they intended to quit in the next 30 days. When comparing female current (n = 116) and former smokers (n = 79), almost half of the female current smokers reported smoking during pregnancy compared to about 20% (n = 16) among female former smokers (p-value = 0.0002). Thus, current female smokers were significantly more likely to smoke during their pregnancy than their former smoker counterparts.

*Multivariate Analyses:* Table 3 shows the results of a logistic regression analysis treating current *versus* former smoking as the binary outcome. Significant predictors of being a current smoker were one’s perception of general health status (comparing perceptions good to fair/poor and perceptions excellent/very good to fair/poor) and smoking policy inside one’s home (allowed/not), after adjusting for a person’s age. Those that perceived their general health to be excellent were more likely to be current smokers than former smokers, with about twice the odds than those with poorer perception of their health (p-value = 0.01). Also those who did not have a no-smoke policy inside their homes were significantly more likely to be current smokers. In fact, such persons had 4 times the odds of being a current smoker than persons who had a no-smoke policy inside their homes (p-value < 0.0001). Model goodness-of-fit was assessed using the Hosmer-Lemeshow test which with a p-value of 0.40 indicated good model fit. Further checks using a receiver operating characteristic (ROC) curve showed that the area under the curve (AUC) of the fitted model was 0.76, significantly greater than 0.50, indicating that the model fit the given data quite well.

## 4. Discussion

Extremely high smoking rates have been reported among American Indian groups since the 1980s [12]. In the early 1990s, a study testing a smoking cessation intervention at California Indian urban and rural healthcare clinics documented a 40% smoking rate among California adult American Indians [3]. Fifteen years later, this study reaffirms the high smoking rate of California’s rural American Indian adults which, at 38%, has remained relatively unchanged over the last decade and a half. The findings of this study show that American Indians are likely smoking at higher rates than the general population, that their smoking rates have remained high for decades, and that there are several factors associated with smoking in this group that may require more targeted intervention to successfully improve cessation rates. American Indian adults’ high-risk behaviors, family and environmental influences are important factors we find to be associated with smoking status.

The factor of environment has complex cultural-undertones. Our study found that American Indian smokers proportionally have less than 50% Indian blood, which raises issues of the influence of assimilation and possible identity conflicts. The Brave Heart study considers the impact of historical trauma to be evident in elevated suicide rates, depression and substance abuse, among others [13]. The differences we observed in suicidal ideation in former and current smokers, compared to never smokers, raise interesting questions regarding the influence of historical trauma, identity issues, and high-risk behaviors. Defined as the cumulative and collective emotional and psychological injury over the life span and across generations, resulting from a group’s past experiences, historical trauma may indeed greatly influence patterns of smoking behaviors among American Indians [14].

It appears that high-risk behavior factors intersect with family factors, as current and former smokers are statistically more likely to drink alcohol, self-report being neglected in childhood and in adolescence and self-report physical abuse in childhood and adolescence than never smokers. Current and former smokers were also more likely to smoke during pregnancy, and have 1–4 others who smoke in the house compared with former and never smokers. The CDC reports that 20% of American Indians reported smoking during the last three months of pregnancy in 2004, a prevalence rate greater than any other ethnic group [15]. This information is important as it paints a picture of abused individuals who practiced risky behaviors, were traumatized by neglect and abuse, and interacted with others in their household who smoke, and exposed their unborn offspring to health risks and possible future smoking addiction.

Finding that only 24% of American Indian smokers reported that they were ready to quit smoking within 30-days (76% reported no intention to quit smoking) points to the need to work with all smokers to bring them into the “ready to quit” phase. It is unlikely that three-quarters of the targeted group who had no intention to quit smoking will listen to and accept cessation messages. Additionally, our data indicate American Indians begin smoking at a younger age, a finding documented in other studies [16], and which influences smoking during adulthood and increases risk for morbidity and mortality. Adaptation of cessation programs (from cessation messages to educational forums) may be necessary to influence changes in attitudes and behaviors, as well as build American Indian smokers’ self-efficacy to quit. Accepting no-smoking messages and participating in smoking cessation programs can only be accomplished if the targeted group hears, understands, and is able to contemplate and act upon the messages directed toward them. One strategy to help American Indian smokers to quit smoking is to educate them about the harmful effects of smoking not only on individuals, but also family members. Messages that alert smokers that American Indian families and communities are at risk for harm, both in the short and long term, are very meaningful. In the American Indian value system, a threat to family is considered more powerful than a threat to the individual. The desire to keep the family and the tribe intact and protect the health of future generations is a strong motivating force among American Indian groups.

Identifying the smoking patterns of current and former smokers and increasing our understanding of the differences in smoking behaviors between populations may help to guide more effective cessation interventions. By isolating specific tobacco use patterns in former smokers we have a clearer view of what messages may be useful as well as needed to influence no-smoking attitudes and behaviors among current smokers. In addition, because rural American Indians were younger and began smoking at a younger age, designing school-based or perhaps elder-youth mentorship programs to educate children about traditional values of health and community and divert them away from the smoking habit are desirable. Community/tribal participatory strategies are recommended for developing meaningful smoking cessation programs that are accessible to rural communities.

### Limitations of the Study

This study provided information on factors associated with smoking among California’s rural American Indian population and further illustrates the unique pattern of smoking, which is likely a result of psycho-social abuse and influences risk-taking behaviors. The subjective nature of one’s perception of “abuse” and “neglect” presents a limitation. For some persons, it is an extreme experience while for others something that others may consider quite mild may be perceived as abuse/neglect. Self-reporting and recall of events, such a physical and sexual abuse in childhood, presents a bias. Although our data was limited to a randomized cohort of rural residents in California and smoking status was self-reported, this study provides important findings about this cohort of current smokers, former smokers and never smokers. Our research further highlights complex issues that need to be addressed in smoking cessation programs, such as low daily cigarette use (less than ½ a pack), high-risk behaviors (smoking during pregnancy and alcohol consumption) and the alarming history of abuse and neglect that the group of smokers and former smokers reported. Additional work is needed to understand the high rates of suicidal ideation among smokers and former smokers. Additional qualitative work is needed to understand the factors associated with the onset and continued use of tobacco products.

## 5. Conclusions

Understanding the factors associated with smoking among American Indians, specifically those factors that are unique and show strong association with smoking, is an important step in cessation. Culturally-sensitive programs will help to bring about a reduction in the extremely high smoking rates and will help to create and implement policy changes and more effective programs to address the problem of high smoking rates among American Indians.

## Figures and Tables

**Table 1.** Characteristics of American Indian Adults by Smoking Status (Current, Former and Never).

**Characteristics**	**Overall (n = 426)**	**Current Smoker (n = 163)**	**Former Smoker (n = 114)**	**Never Smoker (n = 149)**	**p-value**
**Demographic**	Mean (SE)	Mean (SE)	Mean (SE)	Mean (SE)	
Age	44.5 (0.8)	40.2 (1.1)	51.3 (1.6)	43.9 (1.4)	<0.0001*
Annual household income	24,172.4 (1,746)	23,315.1 (2,975.3)	24,179.8 (2,990.9)	25,116.7 (3,101.9)	0.91
	% (n)	% (n)	% (n)	% (n)	
Male	25.4 (108)	28.8 (47)	30.1 (34)	18.1 (27)	0.04*
Marital Status Married or Living with someone Divorced/Separated/Widowed/Single	52.6 (219) 47.4 (197)	59.8 (95) 40.3 (64)	48.2 (54) 51.8 (58)	48.3 (70) 51.7 (75)	0.07
Employed	62.8 (260)	65.0 (104)	56.0 (61)	65.5 (95)	0.23
Education (≥HS degree)	78.2 (333)	77.9 (127)	74.6 (85)	81.2 (121)	0.43
**Health**					
Perception of General Health Fair/poor Good Excellent/Very good	20.7 (88) 38.4 (163) 40.9 (174)	17.8 (29) 37.4 (61) 44.8 (73)	31.6 (36) 41.2 (47) 27.2 (31)	15.5 (23) 37.2 (55) 47.3 (70)	0.002*
Poor Perception of Wellness	26.8 (112)	24.5 (39)	32.5 (37)	24.8 (36)	0.28
BMI <25 25–29.9 30–39.9 40+	29.2 (124) 24.3 (103) 36.1 (153) 10.4 (44)	33.3 (54 24.1 (39) 34.0 (55) 8.6 (14)	25.7 (29) 25.7 (29) 32.7 (37) 15.9 (18)	27.5 (41) 23.5 (35) 40.9 (61) 8.1 (12)	0.25
Limited Activity	28.4 (117)	26.7 (43)	35.8 (39)	24.7 (35)	0.13
**Psycho-Social Characteristics**					
Neglect-Childhood	17.1 (68)	19.6 (29)	22.9 (25)	9.9 (14)	0.02*
Neglect-Adolescence	18.8 (67)	20.9 (29)	26.3 (25)	10.7 (13)	0.01*
Neglect-Adult	13.2 (50)	17.5 (25)	13.9 (14)	8.2 (11)	0.07
Physical abuse-Childhood	15.8 (63)	21.9 (33)	16.4 (18)	8.6 (12)	0.008*
Physical abuse-Adolescence	12.2 (45)	18.1 (26)	13.1 (13)	4.7 (6)	0.003*
Physical abuse-Adult	19.4 (77)	22.7 (34)	21.7 (23)	14.3 (20)	0.16
Sexual abuse-Childhood	14.2 (56)	17.1 (26)	15.2 (16)	10.5 (14)	0.26
Sexual abuse-Adolescence	10.4 (38)	12.3 (18)	8.3 (8)	9.7 (12)	0.57
Sexual abuse-Adult	9.8 (39)	11.7 (18)	9.43 (10)	8.0 (11)	0.57
Suicidal ideation	20.4 (84)	24.2 (38)	24.3 (27)	13.2 (19)	0.03*
Attempted suicide	7.6 (32)	9.3 (15)	8.9 (10)	4.7 (7)	0.26

* significant difference among classifiers based on Chi-square (categorical/binary variables) or F-test based on fitting GLMs (continuous variables) at alpha = 0.05.

**Table 2.** Differences in Smoking Behaviors of Current and Former Smokers among American Indian Adults. n = 277.

**Variables**	**Current Smoker (n = 163)**	**Former Smoker (n = 114)**	**p-value**
**Smoking Behaviors**	Mean (SE)	Mean (SE)	
Age of Smoking Initiation	14.8 (0.3)	14.4 (0.5)	0.52
	% (n)	% (n)	
Smoking during pregnancy None Half a pack or less Half a pack or more	51.1 (45) 38.6 (34) 10.2 (9)	78.8 (52) 16.7 (11) 4.6 (3)	0.002*
**Smoking Environment**	% (n)	% (n)	
Number of smokers in house 0 1 2 or more	5.1 (8) 44.0 (69) 50.9 (80)	70.7 (70) 20.2 (20) 9.0 (9)	<0.0001*
Smoking allowed inside home	54.7 (88)	23.1 (24)	<0.0001*
Visitors allowed to smoke inside home	52.5 (84)	23.5 (24)	<0.0001*

* significant difference between classifiers based on Chi-square test (categorical/binary variables) or two-sample independent t-test (continuous variables) at alpha = 0.05.

**Table 3.** Logistic Regression Showing Predictors of being Current *versus* Former Smoker (n = 244).

	**Estimated β**	**SE (est. β)**	**Estimated OR**	**95% CI (for OR)**	**p-value**
**Age**	–0.05	0.01	0.95	(0.93, 0.97)	<0.0001*
**General Health** (good *vs.* fair/poor) (excellent/very good *vs.* fair/poor)	–0.17 0.53	0.20 0.22	1.2 2.42	(0.57, 2.55) (1.10, 5.37)	0.39 0.01*
**Smoking allowed inside home** (yes *vs.* no)	0.71	0.16	4.10	(2.20, 7.62)	<0.0001*

* Significance based on Chi-square tests from Logistic model fitting at alpha = 0.05.
